# Plasma Membrane Mechanical Stress Activates TRPC5 Channels

**DOI:** 10.1371/journal.pone.0122227

**Published:** 2015-04-07

**Authors:** Bing Shen, Ching-On Wong, On-Chai Lau, Theodosia Woo, Suwen Bai, Yu Huang, Xiaoqiang Yao

**Affiliations:** 1 School of Biomedical Sciences, Faculty of Medicine, the Chinese University of Hong Kong, Shatin, Hong Kong, China; 2 Department of Physiology, Anhui Medical University, Hefei, China; Indiana University School of Medicine, UNITED STATES

## Abstract

Mechanical forces exerted on cells impose stress on the plasma membrane. Cells sense this stress and elicit a mechanoelectric transduction cascade that initiates compensatory mechanisms. Mechanosensitive ion channels in the plasma membrane are responsible for transducing the mechanical signals to electrical signals. However, the mechanisms underlying channel activation in response to mechanical stress remain incompletely understood. Transient Receptor Potential (TRP) channels serve essential functions in several sensory modalities. These channels can also participate in mechanotransduction by either being autonomously sensitive to mechanical perturbation or by coupling to other mechanosensory components of the cell. Here, we investigated the response of a TRP family member, TRPC5, to mechanical stress. Hypoosmolarity triggers Ca^2+^ influx and cationic conductance through TRPC5. Importantly, for the first time we were able to record the stretch-activated TRPC5 current at single-channel level. The activation threshold for TRPC5 was found to be 240 mOsm for hypoosmotic stress and between −20 and −40 mmHg for pressure applied to membrane patch. In addition, we found that disruption of actin filaments suppresses TRPC5 response to hypoosmotic stress and patch pipette pressure, but does not prevent the activation of TRPC5 by stretch-independent mechanisms, indicating that actin cytoskeleton is an essential transduction component that confers mechanosensitivity to TRPC5. In summary, our findings establish that TRPC5 can be activated at the single-channel level when mechanical stress on the cell reaches a certain threshold.

## Introduction

Proteins embedded in the lipid bilayer are constantly exposed to the mechanical forces exerted on the bilayer [1]. External mechanical forces acting on the bilayer change the transverse pressure profile, and directly transduce the pressure to the embedded protein by lipid-protein interactions. In the case of an ion channel, alterations in bilayer tension or curvature causes a hydrophobic mismatch in the protein-lipid interface, causing the channel protein to adopt a new conformation that favors either an open or close conformation status of the conducting pore [2]. Alternatively, the cytoskeleton or extracellular matrix can be the primary force sensor which can transduce force to a tethered ion channel by displacement, leading to conformational change of the channel [2–4]. In addition, force sensitive enzymes may generate second messengers that modulate ion channel activity, thereby conferring the mechanosensitivity to that channel [2–4]. Regardless of the kind of mechanical stress or the signal transduction pathway, an ion channel is deemed mechanosensitive when its activity is altered in response to mechanical stimuli. Mechanosensitive channels transduce mechanical forces into electrical signals and are essential for various processes ranging from cell osmotic regulation to organismal sensory perception [2, 5]. The bacterial MscM, MscS and MscL open in response to osmotic shock, thus allowing release of cytoplasmic solutes. In yeast, similar function is performed by TRPY to regulate vacuolar osmotic balance [6]. The *C*. *elegans* MEC4 forms the ion conducting pore within a mechanosensitive complex to sense tactile stimuli [7]. In mammalian neuron, the mechanosensitive TREK-1 conducts K^+^ ions to set resting membrane potential [8]. Recently, Piezo was identified as mechanosensitive channel that is essential for sensing noxious pressure in *Drosophila* and mammalian cells [9, 10].

Since the cloning and characterization of the first member of the Transient Receptor Potential (TRP) channel family, it has been well established that TRP channels play fundamental roles in sensory biology [11]. Indeed, TRPC1, TRPC6, TRPM3, TRPM4, TRPV1, TRPV2, TRPV4 and TRPA1 have been reported to be involved in cellular mechanosensory transduction [12–19]. However, in order to assess whether a given TRP channel is mechanosensitive, it is necessary to employ comprehensive pharmacological and electrophysiological methods to verify it. In this regard, increased channel activity after applying force to the channel embedded in cell membrane is crucial to demonstrate the mechanosensitivity of the channel [20].

TRPC5 is a polymodal channel that is enriched in neuronal cells and also localizes to the aortic baroreceptor termini, which are sensory neuronal termini for blood pressure detection [21]. In addition to being sensitive to a variety of lipids and lipid derivatives [22], TRPC5 can be activated by a bilayer perturbing isoflavonoid genistein [23]. Interestingly, genistein and structurally similar derivatives induce local thinning of lipid bilayer [24]—also an outcome of membrane stretch. Given its expression profile and functional properties, we asked whether TRPC5 functions as a mechanosensitive channel. To answer this question, we used live cell Ca^2+^ imaging and electrophysiology to characterize the mechanosensitivity of TRPC5 channels. Consistent with the findings reported in a previous study [25], but by utilizing independent reagents and new approaches, we confirmed that hypotonic membrane stretch activates TRPC5, in a manner that is independent of phospholipase C. Furthermore, we directly applied force to the TRPC5-containing membrane patch and recorded stretch activation of TRPC5 at single-channel level. Our results indicate that mechanical stress induced by either hypoosmolarity or micropipette suction stimulates TRPC5 activity, and that the stimulatory mechanism is dependent on actin filaments.

## Materials and Methods

### Cell culture and cDNA expression

The mouse TRPC5 cDNA (NM_009428.2) and the mouse TRPC6 cDNA (NM_013838.2) were gifts from L. Birnbaumer (NIH, USA), and were subcloned into either pcDNA3 (TRPC5-pcDNA3 and TRPC6-pcDNA3) or a bicistronic expression vector pcDNA6-IRES-GFP (TRPC5-I-GFP). The C-terminally truncated form ΔC-TRPC5 lacks the last 9 amino acid residues, and was cloned from the mouse TRPC5 cDNA by PCR. The cDNA of ΔC-TRPC5 was subcloned into pcDNA6. Human embryonic kidney (HEK293) cells were cultured in DMEM, and Chinese Hamster Ovary (CHO-K1) cells were cultured in F12/HAM medium. The culture media were supplemented with 10% FBS. Stable HEK293 cell lines containing pcDNA3, TRPC5 or ΔC-TRPC5, and stable CHO-K1 cell lines containing pcDNA6-IRES-GFP or TRPC5-I-GFP were generated respectively. To generate the stable cell lines, ~6x10^5^ cells were transfected with 4 μg of respectively plasmid DNA using Lipofectamine 2000 (Invitrogen), and subsequently cultured in DMEM with appropriate antibiotics, 500 μg/mL G418 for pcDNA3 construct and 3 μg/ml blasticidin for pcDNA6, to select for stably transfected cells. For the stable CHO-K1 cell lines, GFP-positive colonies were selected during the 2nd, 3rd and 4th passage for continuous culture. Cells were grown in selection medium for at least 10 passages before being used for experiments.

### Preparation of the TRPC5-blocking antibody T5E3 and preimmune IgG

T5E3 antibody was raised in rabbits as described [23, 26]. Briefly, a peptide corresponding to TRPC5 putative pore-region (CYETRAIDEPNNCKG; E3 peptide) was used to immunize rabbits. Antiserum was collected. IgG was purified from the T5E3 antiserum using a HisTrap protein G column (GE Healthcare). The T5E3 antibody was further purified from the IgG by an affinity column prepared with E3 peptide-conjugated SulfoLinked Coupling Resin (Thermo Scientific). Control IgG was purified from serum of pre-immunized rabbits using HisTrap protein G column. To inhibit TRPC5, cells were pretreated with T5E3 (15 μg/ml) or pre-immune serum IgG (15 μg/ml) for 1 hour.

### Immunoblots detection of TRPC5 in lysates

The cultured cells were trypsinized and washed three times with ice-cold PBS. The cell pellet was collected by centrifugation at 1600 rpm, followed by trituration in 500 μl ice-cold freshly prepared lysis buffer (20 mM Tris-HCl, 150 mM NaCl, 1mM EGTA, 0.5% Triton X-100, pH7.3). The lysate was centrifuged at 12,000 rpm for 30 min at 4°C. The supernatant were collected and the protein concentration was determined by Bradford assay. Subsequently, equal amounts of protein were boiled in SDS-PAGE loading buffer and loaded at ~80 μg into each lane of polyacrylamide gel and separated by a 7.5% SDS-PAGE gel. Proteins were then transferred to a PVDF membrane, which was first blocked by 5% non-fat milk and 0.1% Tween-20 in PBS. Proteins were blotted with the primary antibodies against anti-TRPC5 antibody (1:200; Alomone Lab) or anti- β-tubulin antibody (1:100; Santa Cruz Biotechnology). Immunodetection was accomplished with secondary antibody conjugated with horseradish peroxidase, followed by reaction with ECL Western blotting detection system (Amersham).

### [Ca^2+^]_i_ measurement

**Fig 1:**
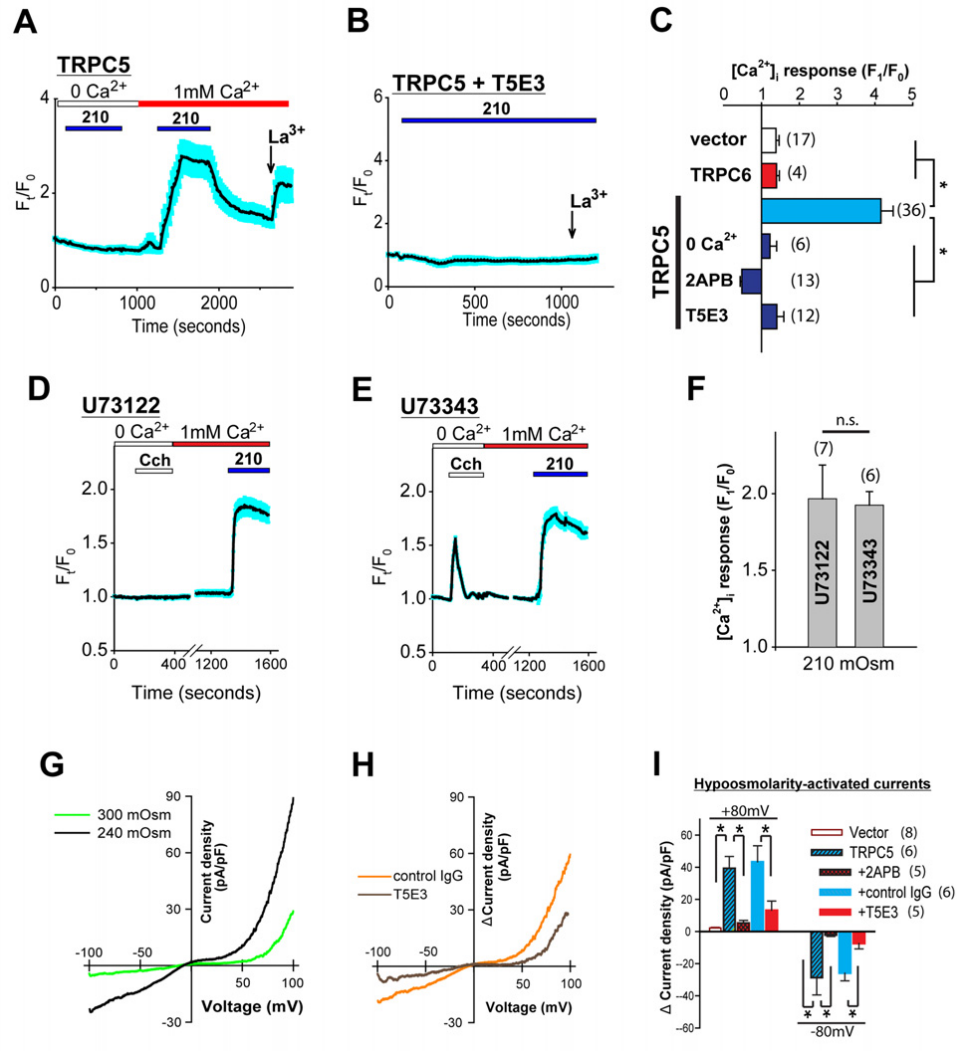
Hypoosmolarity induces Ca^2+^ influx and whole-cell currents through TRPC5. (A-B) representative time-series traces showing [Ca^2+^]_i_ responses to hypotonicity (210 mOsm) in TRPC5-HEK cells. *Blue bars* on top indicate duration of hypoosmolarity. Lanthanum (La^3+^, 100 μM) directly potentiates TRPC5 activity and was used to show functionality of the express TRPC5. T5E3 (4 μg/ml) is TRPC5-specific blocking antibody and was applied extracellularly to inhibit channel activity (B). (C) quantification of [Ca^2+^]_i_ response to 210 mOsm at different conditions. (D-E), representative time-series traces showing [Ca^2+^]_i_ responses to carbachol (Cch, 100 μM) and 210 mOsm when TRPC5-HEK cells were treated by 10 μM of U73122 (D) or U73343 (E) for 30 minutes prior to the recordings. (F) quantification of [Ca^2+^]_i_ response to 210 mOsm as in (D and E). (G) whole-cell *I-V* relationships of a representative TRPC5-HEK cell under isotonic (300 mOsm) and hypotonic (240 mOsm) conditions. (H) *I-V* relationships of hypoosmolarity-activated currents in cells pretreated with preimmune IgG (*control IgG*, 15 μg/ml) or T5E3 (15 μg/ml). The curves were obtained by subtracting the basal currents at isotonic condition from that at hypotonic condition. (I) summary of data showing hypoosmolarity-activated whole-cell current density at ±80 mV in vector stably-transfected HEK293 cells, TRPC5-HEK cells and TRPC5-HEK cells treated with 2APB (75 μM), preimmune IgG (*+control IgG*) and T5E3. Values represent mean ±SEM.

The concentration of intracellular Ca^2+^ ([Ca^2+^]_i_) was measured as described previously [23]. Briefly, cells were seeded onto poly-L-lysine-coated glass discs one day before [Ca^2+^]_i_ measurement. The cells were loaded for 1 h in dark with 5 μM Fluo-3/AM and 0.02% pluronic F-127 in culture media, before mounting onto a microscope chamber containing an isotonic bath solution (in mM: 65 NaCl, 5 KCl, 1 CaCl_2_, 1 MgCl_2_, 10 HEPES, pH7.4; osmolarity calibrated to 300 mOsm with ~140 mM mannitol). Hypotonic bath solutions contained identical ionic concentrations to isotonic bath solution, and the osmolarity calibrated to different values by varying the concentration of mannitol. Normal physiological saline solution (NPSS) contained in mM: 140 NaCl, 5 KCl, 2 MgCl_2_, 1 CaCl_2_, 10 Glucose, 10 HEPES, pH 7.4. Fluorescence [Ca^2+^]_i_ signals were recorded by InCyt Basic Fluorescence Imaging. Data shown in Fig 1D and 1E were acquired by Nikon T200 fluorescence microscope. Changes in [Ca^2+^]_i_ were displayed as F_t_/F_0_— a ratio of real-time fluorescence (F_t_) relative to the intensity at the beginning (F_0_) of the experiment. Maximal change in [Ca^2+^]_i_ was presented as ratio F_1_/F_0_. All experiments were performed at room temperature (~ 23°C).

### Electrophysiology

Whole-cell voltage clamp and single channel recordings were performed using an EPC9 patch clamp amplifier (HEKA, Germany). For whole-cell recording, the holding potential was 0 mV, and I-V relationship was obtained using a ramp protocol from −100 mV to +100 mV over a duration of 500 ms. When >15% change in current amplitude was observed after applying hypotonic stress (regarded as the onset of response), currents at ±60 mV were sampled every 10 seconds continuously for a further 10 minutes. For some experiments, the cells were pretreated with T5E3 (15 μg/ml) or pre-immune serum IgG (15 μg/ml) for 1 hr, or with 2-APB (75 μM) or Gd^3+^ (20 μM) for 15 min before the recording. The whole-cell current was normalized by cell capacitance (value reported by the HEKA Pulse software) into current density (pA/pF). For single-channel recordings, a U-shaped water-filled hydraulic pressure system was used to generate pressure. When needed, the patch pipettes were backfilled with an anti-TRPC5 blocking antibody T5E3 (15 μg/ml) or pre-immune serum IgG (15 μg/ml) using a two-step protocol as described [27, 28]. Single-channel recordings were performed continuously for ~15 min. T5E3 inhibition was observed in 5–10 min after gigaseal. All currents were sampled at 50 kHz and filtered at 5 kHz. The junction potential was determined by the software IGOR with Patcher's Power Tools, Version 2.07 10.10 2005 (Dr. Francisco Mendez; Max-Planck-Institut für biophysikalische Chemie), and was calculated to be approximately +16.66 mV for the combination of pipette solution (see below for ionic composition) and bath solution NPSS. The whole-cell and single-channel current data were analyzed with PulseFit and TACFit software, respectively. All experiments were performed at room temperature. Solutions for patch-clamp experiments include: (1) In whole-cell recordings, the pipette (intracellular) solution contained in mM: 130 Cs-aspartate, 2 MgCl_2_, 5 Na_2_ATP, 5.9 CaCl_2_, 10 EGTA, 10 HEPES, pH 7.4 with CsOH. Free Ca^2+^ was calculated to be ~200 nM by the Maxchelator program (http://maxchelator.stanford.edu). The bath solution was either the isotonic solution or the hypotonic solution, with NaCl being replaced by equal molar of Na-aspartate. (2) In cell-attached channel recordings, the pipette (extracellular) solution contained in mM: 140 Cs-aspartate, 5 CsCl, 1 MgCl_2_, 1 CaCl_2_, 10 HEPES, pH 7.4 with CsOH. The bath solution for the cell-attached recordings was NPSS. (3) For determining channel conductance in cell-attached mode, high K^+^ bath solution was used, which contained in mM: 130 K-aspartate, 5 NaCl, 1 MgCl_2_, 1 CaCl_2_, 10 glucose, 10 HEPES, pH 7.4 with KOH. For experiments with BAPTA-AM treatment, cells were pretreated with 10 μM BAPTA-AM in Ca^2+^ free NPSS for 40 minutes before cell-attached recordings. For experiments with cytochalasin D treatment, cells were pretreated with 5 μM cytochalasin D for 30 minutes in NPSS, and subjected to single-channel recording within 30 minutes.

### Data analysis and presentation

Data from [Ca^2+^]_i_ measurement were plotted as traces showing mean ± SEM of F_t_/F_0_ from ≥20 cells per experiment. Current-voltage curves in electrophysiology were single representative recording from multiple experiments. Bar charts show data in mean ± SEM of the measured values from multiple experiments. Number of experiments (n) was indicated in *bracket* at the bar apex or next to the legend label. Student’s t-test was used for statistical comparison, with probability *p*<0.05 (*) deemed to be a significant difference. The absence of significant difference (*p*≥0.05) was denoted as "n. s.".

## Results

### Hypoosmotic stress activates TRPC5

It is known that an acute decrease in the osmolarity of the bath solution elicits cell swelling accompanied by an increase in plasma membrane tension [29, 30]. Thus, hypotonic shock induced by low osmolarity bath solution can be used as a stimulus to investigate mechanosensitive components on plasma membrane. Previous studies have demonstrated that hypotonic shock increased Ca^2+^ influx and whole-cell currents via TRPC5 [25, 31]. To test whether TRPC5 is a mechanosensitive channel, we sought to verify whether TRPC5 activity is stimulated by hypoosmotic stress using independent reagents and approaches. We established a stable HEK293 line overexpressing mouse TRPC5 (TRPC5-HEK) (Figure A in S1 Fig), and loaded these cells with a fluorescence Ca^2+^ indicator, Fluo3, to measure the change in [Ca^2+^]_i_. We bathed the cells in isotonic solution (300 mOsm) and acutely changed the solution to hypotonic solution (210 mOsm) within 30 seconds (Figure B-C in S1 Fig). The cells swelled gradually within 2–3 minutes after changing bath to hypotonic solution (Figure B in S1 Fig). Accompanying the swelling, [Ca^2+^]_i_ of individual cells increases as indicated by Fluo3 fluorescence (Figure C in S1 Fig).

The same hypotonic stress (210 mOsm) was unable to elicit significant [Ca^2+^]_i_ change in vector stably-transfected cells (Figure A in S2 Fig), which displayed intact Ca^2+^ store release upon stimulation by carbachol (Cch) (Figure A in S2 Fig), a G-protein-coupled receptor (GPCR) agonist to acetylcholine receptor. Contrary to the lack of hypotonic stress induced Ca^2+^ response in vector stably-transfected cells, TRPC5-HEK cells showed robust [Ca^2+^]_i_ elevation when hypotonic stress was applied in the presence of extracellular Ca^2+^ (Fig 1A). [Ca^2+^]_i_ decreased upon restoring bath to isotonic solution. Lanthanum (La^3+^, 100 μM), which directly potentiates TRPC5 activity [32], was applied before the end of the experiment and elicit [Ca^2+^]_i_ increase (Fig 1A), indicating functionality of plasma membrane TRPC5. The [Ca^2+^]_i_ rise in TRPC5-HEK is not due to Ca^2+^ release, because depleting endoplasmic reticulum Ca^2+^ stores with thapsigargin did not prevent the hypoosmolarity-induced [Ca^2+^]_i_ rise (Figure B in S2 Fig). The hypoosmolarity-induced [Ca^2+^]_i_ rise was abolished when 2APB (75 μM), a TRPC5 inhibitor [33, 34], was present in the bath (Fig 1C). These results are consistent with a previous study showing hypoosmotic activation of TRPC5 [25]. To demonstrate that the response is indeed specific to TRPC5, we utilized the blocking antibody T5E3, which specifically targets the extracellular turret of TRPC5 protein [23, 26], and found that T5E3 effectively blocked the hypotonic [Ca^2+^]_i_ rise (Fig 1B and 1C). These data demonstrate that the hypotonic shock causes cell swelling to activate TRPC5 in the plasma membrane, leading to Ca^2+^ influx.

It is known that activation of GPCRs increases TRPC channel activity [11, 35]. Some GPCRs are mechanosensitive [36], thus they are potentially capable of activating the downstream phospholipase C (PLC) and G-proteins, and thereby modulating TRPC channel activity. TRPC5 can be activated by either hypoosmolarity or whole-cell inflation by patch pipette pressure [25]. In addition, pharmacological inhibition of PLC by U73122 did not reduce the whole-cell currents in TRPC5 overexpressing cells in response to hypoosmotic shock [25]. However, PIP_2_, the PLC substrate, was found to be required for the hypoosmotic activation of TRPC5 [25]. A recent study by Jemal *et al*. showed that when TRPC5 is co-expressed with the PLC-coupled type 1 histamine receptor, U73122 treatment reduced, but did not completely abolish, the percentage of cells that displayed Ca^2+^ response to hypoosmotic shock [31]. It was proposed that in addition to the hypoosmotic shock activation of the plasma membrane resident TRPC5, stimulation of the overexpressed histamine receptor by hypoosmotic shock caused a PLC-dependent cytosolic Ca^2+^ mobilization that facilitated plasma membrane insertion of TRPC5 [31]. It is not known whether the Ca^2+^ response of TRPC5 to hypoosmolarity is a result of a mechanosensitive GPCR-PLC pathway endogenous to HEK293 cells. PLC can be activated by G_q_-coupled GPCR agonist, for example carbachol (Cch), to produce IP_3_ which triggers intracellular Ca^2+^ store release. In TRPC5-HEK cells, treatment with U73122 effectively blocked the Ca^2+^ release elicited by Cch (100 μM) but not the Ca^2+^ influx in response to hypoosmolarity (Fig 1D and 1F). In addition, treatment with the inactive analog, U73343, resulted in comparable Ca^2+^ response to hypoosmolarity (Fig 1E and 1F), suggesting that such TRPC5 activity is independent of PLC activation. In addition, the lack of hypoosmolarity response for TRPC6 (Fig 1C and Figure C in S2 Fig), which is also a PLC-activated channel, further confirms that hypotonicity fail to activate the endogenous PLC pathway.

We also examined the effect of T5E3 blocking antibody to the whole-cell currents of TRPC5-HEK in response to hypoosmolarity. Previous studies showed that TRPC5-overexpressing cells exhibited larger whole-cell currents at hypoosmolarity [25, 31]. Here, we first verified whether such response was conserved in the TRPC5-HEK stable cells. In a whole-cell configuration, we buffered the intracellular free Ca^2+^ to 200 nM with EGTA to minimize the impact of intracellular Ca^2+^ fluctuation which has been shown to alter the activity of TRPC5 [37]. When the isotonic extracellular bath solution was replaced with a hypotonic solution, the whole-cell currents of TRPC5-HEK cells increased and reached plateau at around 400 seconds after bath exchange (Figure D in S2 Fig). Current-voltage relationship of the whole-cell currents at hypoosmolarity displayed double rectifications (Fig 1G), which is a characteristic of homomeric TRPC5 channels [32, 38]. We applied channel inhibitor 2APB or T5E3 to the bath solution, and both were able to suppress the hypoosmolarity-induced current elevations (Fig 1H and 1I). Together with the results from [Ca^2+^]_i_ imaging experiments, these data demonstrate that hypotonic cell swelling activates TRPC5 on the plasma membrane.

### Gadolinium does not inhibit TRPC5 response to hypoosmotic stress

Previous studies have shown that the trivalent ion gadolinium (Gd^3+^) blocks multiple mechanosensitive channels [20, 39]. On the other hand, there is another class of mechanosensitive channels that are insensitive to Gd^3+^ [40–42]. TRPC5 activity is reportedly potentiated by Gd^3+^ and La^3+^ via the glutamate residue in the pore-loop region [32]. Therefore, we tested the effect of Gd^3+^ on TRPC5-mediated [Ca^2+^]_i_ rise in response to hypoosmolarity. After the [Ca^2+^]_i_ rise in response to hypoosmolarity reached its plateau, 20 μM GdCl_3_ was then added to the bath, which elicited a further elevation of [Ca^2+^]_i_ (Figures A-B in S3 Fig). Such additive effect suggests that Gd^3+^ does not block the TRPC5 response to hypoosmolarity, and that the mechanism of hypoosmolarity activation is independent of Gd^3+^ potentiation.

### Pipette suction activates single TRPC5 channels

**Fig 2:**
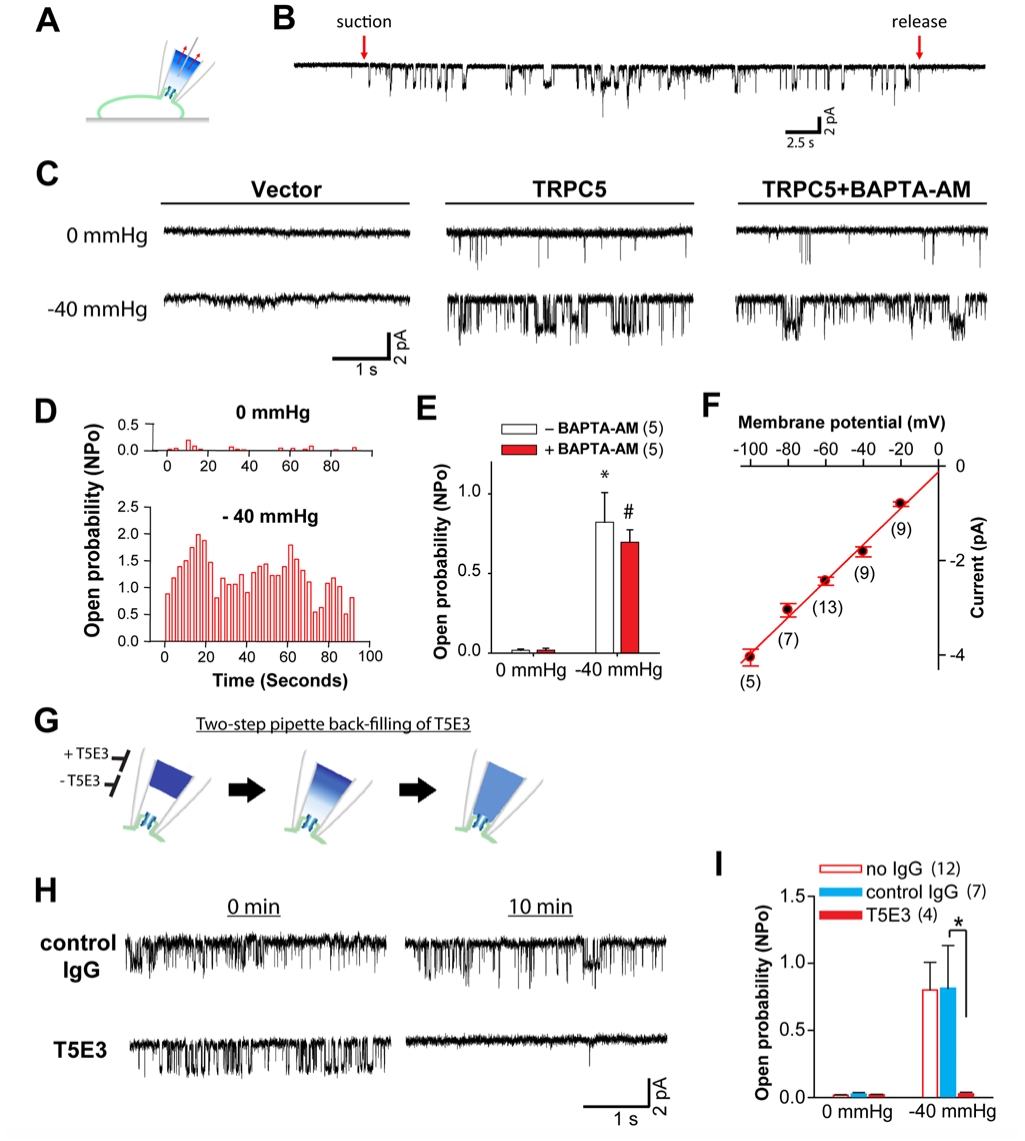
Pipette pressure activates TRPC5 on single-channel membrane patch. (A) schematic diagram depicting single-channel current measurement in TRPC5-expressing CHO-K1 cells. Membrane stretch was elicited by applying suction through the patch pipette, as indicated by *red arrow*. (B) a representative cell-attached recording (n = 11) of TRPC5-expressing CHO-K1 cell showing changes in channel activity when -40 mmHg pipette pressure was applied (*suction*) and subsequently released (*release*). The pipette holding potential was -60 mV. (C) representative traces showing single-channel activities in vector transfected (*Vector*) and TRPC5-expressing (*TRPC5*) CHO-K1 cells at 0 mmHg and -40 mmHg pipette pressure with cell-attached configuration. Channel activities were also recorded in TRPC5-expressing cells pretreated with 10 μM BAPTA-AM to buffer cytosolic Ca^2+^ fluctuation (*TRPC5+BAPTA-AM*). The pipette holding potentials were -60 mV. (D) channel open probability (NPo) values over time for the stretch-activated channel under *0 mmHg* and *-40 mmHg*. Shown are analyses from the same cell-attached patch applied with the indicated pipette pressures for 90 seconds. (E) quantification of the single-channel open probabilities (NPo) of the channel activities recorded on TRPC5-expressing CHO-K1 cells as in (C). (F) single-channel *I-V* relationships of the stretch-activated channels in cell-attached configuration. The bath solution was 130 mM K^+^ solution (*High K* ^*+*^, *red circle*) for dissipating the membrane potential. The slope conductance is 39 ± 2 pS. (G) schematic diagram showing the two-step backfilling protocol. Patch pipettes were backfilled with T5E3 (15 μg/ml) using a two-step protocol. T5E3 eventually diffused to pipette tip to inhibit TRPC5. Only the patched membrane is depicted for simplicity. Recordings were performed with cell-attached configuration. (H) representative traces showing the stretch (-40 mmHg)-activated channel in the presence of preimmune IgG (15 μg/ml) or T5E3 (15 μg/ml) immediately (*0 min*) and 10 minutes (*10 min*) after gigaseal formation. The pipette holding potentials were -60 mV. (I) summary of single-channel open probabilities (NPo) as in (H).

Single-channel patch clamp recordings allow characterization of biophysical properties for individual ion channels. In this technique, mechanical stress can be applied directly to the channel-containing membrane patch by altering the pressure inside the micropipette (Fig 2A) [20, 43]. Pipette suction increases the curvature and tension of the patched membrane [44, 45]. We asked whether such mechanical stress applied locally to the membrane would activate the embedded TRPC5. By connecting the patch pipette to a U-shape water column, we can set the pipette pressure to defined pressure values and record channel activity at single-channel resolution.

Although we did not observe Ca^2+^ influx in control HEK293 cells when challenged by hypoosmolarity, we frequently encountered endogenous large-current channels when we applied negative pipette pressure to HEK293 cells. Because patch clamp experiments measure currents across the patched membrane that can be carried by any available ions in the extra- and intra-cellular solutions (for instance, Cs^+^, K^+^, or Na^+^), these endogenous mechanosensitive channels may mask the channel activity of TRPC5 in single-channel experiments. Therefore, for single-channel studies, we chose to use CHO-K1 cells, in which we rarely recorded mechanosensitive currents. We then established a CHO-K1 cell line stably overexpressing TRPC5 (Figure A in S1 Fig).

In the cell-attached (c/a) mode (Fig 2A), negative pressure (suction) in the pipette at −40 mmHg dramatically increased a TRPC5-like channel activity in TRPC5-overexpressing CHO-K1 cells (Fig 2B and 2C). The channel open probability (NPo) increased from a control value of 0.02 ± 0.01 to the values of 0.82 ± 0.19 at −40 mmHg (Fig 2D and 2E). However, in control cells transfected with an empty vector, the same pressure steps failed to elicit comparable channel activity (Fig 2C). Since TRPC5 activity can be potentiated by cytosolic Ca^2+^ elevation [37], we asked how the channel activity in response to pressure would change if the cytosolic Ca^2+^ fluctuation is buffered. We treated the cells with 10 μM BAPTA-AM before the recordings to chelate cytosolic Ca^2+^, and found that the channel open probability increased significantly when −40 mmHg was applied (Fig 2C and 2E), indicating that the channel response to pressure is not mediated by cytosolic Ca^2+^ elevation. We also attempted to perform excised patch recording to test whether TRPC5 is responsive to pressure in the absence of cytosolic components. After performing cell-attached recordings, we excised the membrane patch that contained TRPC5 channel and tested for channel activity. Although it was technically challenging to apply pressure to the excised patch without disrupting the seal or the patched membrane, we observed that in some excised patches, TRPC5 channel activity increased after applying pressure (S4 Fig).

To estimate the single-channel slope conductance of this pressure-activated channel, current amplitudes were measured at different clamping potentials while the cells were bathed in a 130 mM extracellular K^+^ solution to dissipate the resting membrane potential. The slope conductance of this stretch-activated channel in c/a patch was estimated to be 39 ± 2 pS (Fig 2F), which was close to the reported value of 38 ± 3 pS for homomeric TRPC5 channels [32, 46].

We further verified this mechanical stress-activated channel by apply T5E3 blocking antibodies to the patch pipette via a two-step backfilling method (Fig 2G). Shortly after the formation of a gigaseal, suction was able to elicit TRPC5-like channel response (Fig 2H). After 5–10 minutes to allow diffusion of T5E3 to the pipette tip, the same suction failed to elicit channel activity (Fig 2H and 2I). In contrast, back-filling the pipettes with preimmune control IgG was found to have no effect on the suction-induced response (Fig 2H and 2I). Together, these results demonstrate that TRPC5 can be activated by mechanical stretch applied to the plasma membrane.

### Graded response of TRPC5 to mechanical stress

**Fig 3:**
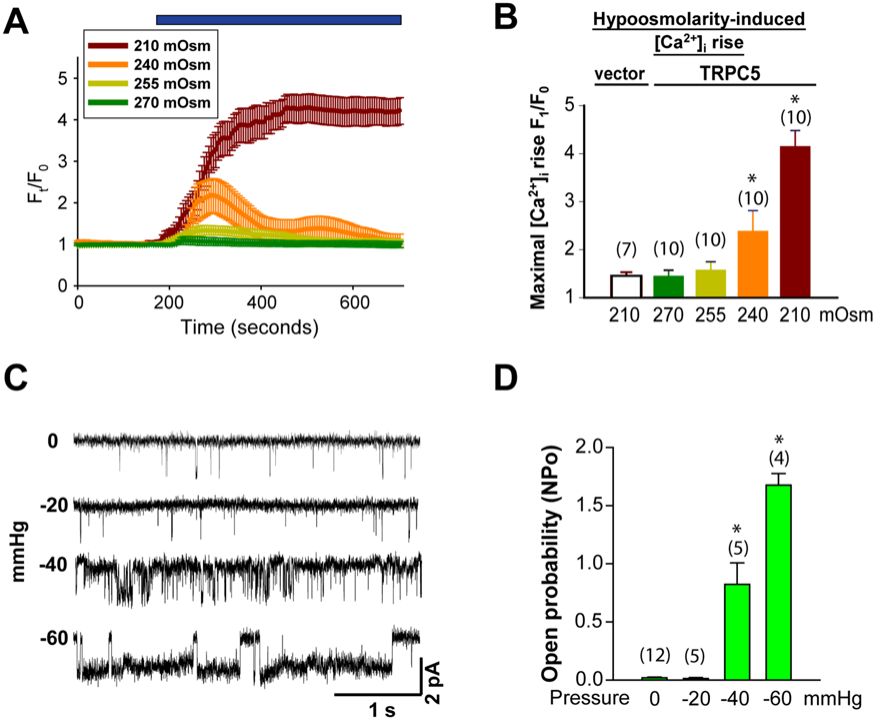
Threshold of TRPC5 mechanosensitivity. (A) representative time-series traces showing [Ca^2+^]_i_ in TRPC5-expressing HEK293 cells in response to different osmolarities. Blue bar on top indicated duration of hypoosmolarity. (B) quantification of the [Ca^2+^]_i_ response at different osmolarities. *, *p<0*.*05* compared to *270 mOsm*. (C) representative traces showing the stretch-activated channel under different pipette pressures from a single cell-attached patch of TRPC5-expressing CHO-K1 cell. (D) quantification of single-channel open probabilities (NPo) of under different pipette pressure as in (C). *, *p<0*.*05* compared to *0 mmHg*.

Many mechanosensitive channels exhibit a pressure threshold, across which the channel is activated [47]. We challenged the TRPC5-expressing cells with bath solutions of different osmolarities. The results showed that significant [Ca^2+^]_i_ rise occurred at or below 240 mOsm (Fig 3A and 3B). Similarly, on single-channel patch clamp recordings, −20 mmHg had no effect on TRPC5 NPo, while −40 mmHg and −60 mmHg significantly increased NPo (Fig 3C and 3D). Therefore, the single-channel pressure threshold necessary to activate TRPC5 occurs between −20 and −40 mmHg. These results indicate that TRPC5 activity is significantly enhanced only when plasma membrane tension reaches a permissive threshold.

### TRPC5 response to hypoosmotic stress is dependent on actin filaments

**Fig 4:**
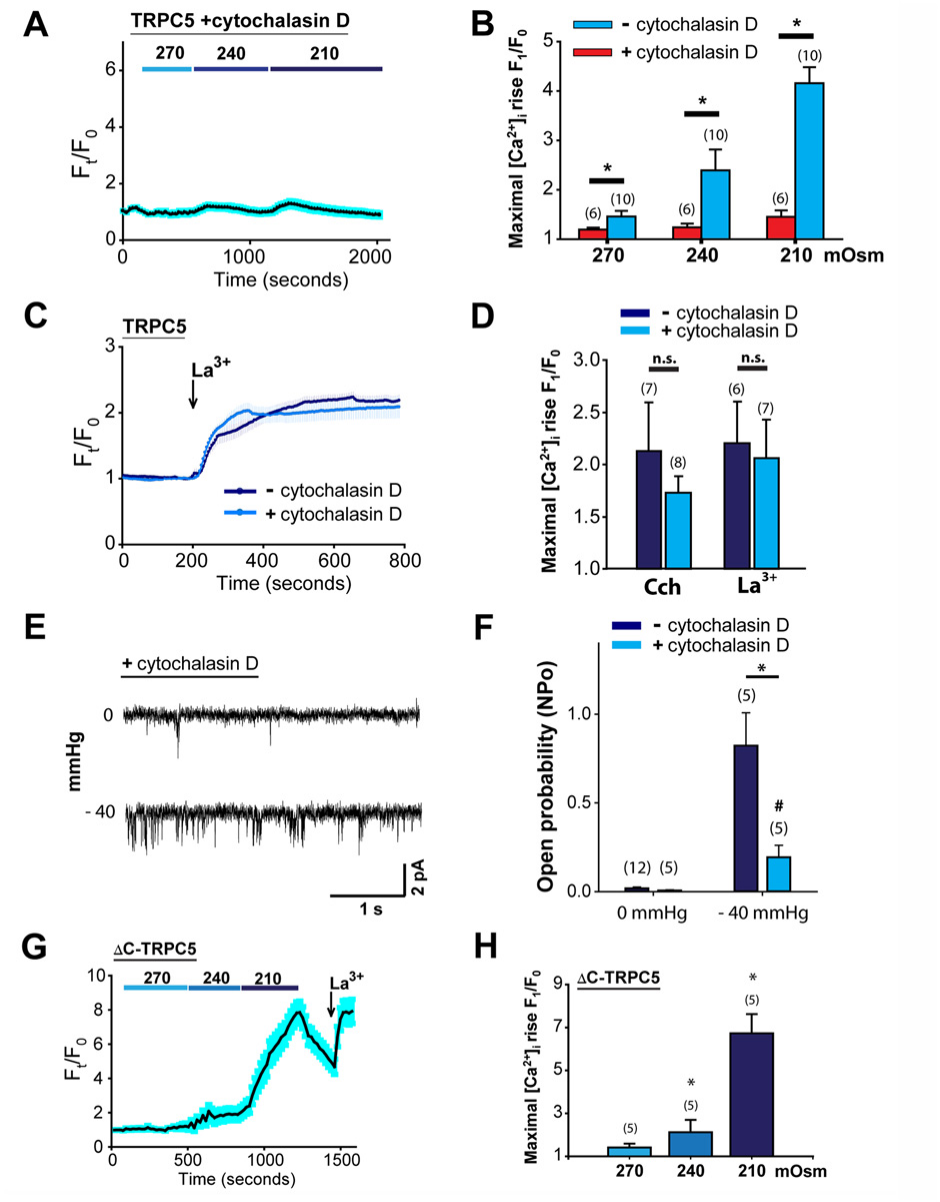
Actin filament is essential to TRPC5 mechanosensitivity to hypoosmotic stress. (A) a representative time-series trace showing [Ca^2+^]_i_ responses to different osmolarities in TRPC5-expressing HEK293 cells that were pretreated with 25 μM cytochalasin D for 45 min. (B) summary showing effect of 25 μM cytochalasin D treatment on the [Ca^2+^]_i_ responses to hypoosmolarity. (C) representative time-series traces showing [Ca^2+^]_i_ responses to 100 μM LaCl_3_ (La^3+^, *arrow* indicates time of addition) in cells that were treated with or without 25 μM cytochalasin D. (D) summary of the [Ca^2+^]_i_ responses to 100 μM carbachol (*Cch*) or 100 μM LaCl_3_ (*La* ^*3+*^). *n*.*s*. denotes "not significant". E, a representative cell-attached patch of TRPC5-expressing CHO-K1 cells pretreated with 5 μM cytochalasin D at 0 mmHg and -40 mmHg pipette pressure. F, quantifications of channel open probability (NPo) from patches with or without cytochalasin D treatment. NPo of cytochalasin D treated patches is significantly lower compared to the untreated at -40 mmHg (*, *p<0*.*05*), and is significantly higher compared to that at 0 mmHg (*#*, *p<0*.*05*). (G) representative time-series trace showing [Ca^2+^]_i_ response to different osmolarities in HEK293 cells expressing ΔC-TRPC5, a truncated form lacking the C-terminal PDZ-binding motif. (H) summary of [Ca^2+^]_i_ responses to different osmolarities in ΔC-TRPC5-expressing HEK293 cells. *, *p<0*.*05* compared to *270 mOsm*.

Cortical actin forms a polymerized network underneath the plasma membrane to provide rigidity and integrity to the lipid bilayer. This array of filaments minimizes lateral stretch and protects the bilayer from sudden changes in bilayer tension, which may lead to rupture of the membrane. Hence, the presence of cortical actin may limit the transmission of experimentally applied force to mechanosensitive components within the lipid bilayer. On the other hand, in the tethered model, actin filaments may convey force to a tethered ion channel, thereby conferring the mechanosensitivity to the channel [3, 4]. To test whether cortical actin filaments modulate the hypoosmolarity-induced TRPC5 activity, we treated the TRPC5-expressing cells with cytochalasin D (25 μM) to disrupt actin polymerization prior to hypotonic stimulation. Interestingly, cytochalasin D treatment abolished the sensitivity of the channel to hypoosmolarity (Fig 4A and 4B), but had no effect on response of TRPC5 to La^3+^ and Cch (Fig 4C and 4D). These data suggest that although disrupting actin polymerization blunts TRPC5 sensitivity to hypotonic membrane stretch, this manipulation does not affect channel activation via plasma membrane delimited mechanisms. We also tested the effect of cytochalasin D treatment on the single-channel stretch response of TRPC5. We treated TRPC5-overexpressing CHO-K1 cells with cytochalasin D (5 μM) and performed cell-attached single-channel recordings. Whereas pipette suction (−40 mmHg) elicited significant channel openings under cytochalasin D treatment (Fig 4E), the open probability was drastically decreased by ~75% compared to untreated control (Fig 4F). Therefore, actin filaments are crucial for TRPC5 response to mechanical stress.

One possible way for actin filaments to regulate TRPC5 sensitivity to mechanical stress is by tethering to the channel's cytosolic domains. TRPC5 contains a PDZ-binding domain at the C-terminus. It has been shown that the cytoskeletal-anchoring protein NHERF/EBP50 can physically couple to TRPC5 via the C-terminal PDZ-binding motif [48, 49]. Since EBP50 interacts with actin via ezrin, disruption of TRPC5-EBP50 coupling might prevent actin regulation of TRPC5 upon mechanical stress. Therefore, we constructed a truncated TRPC5 (ΔC-TRPC5) in which the C-terminal 9 amino acid residues that contain the PDZ-binding motif (VTTRL) were removed. However, the truncation did not prohibit the channel response to hypoosmolarity (Fig 4G and 4H), suggesting that the C-terminal PDZ-binding domain of TRPC5 is not involved in cytoskeleton tethering for mechanosensitivity.

## Discussion

In the present study, we characterized the mechanosensitive properties of TRPC5. We utilized two methods to apply mechanical force to the plasma membrane: 1) acute cell swelling induced by hypoosmolarity; and 2) suction force applied via patch pipette to increase tension of the patched membrane. We found that either manipulation led to an increase in channel activity as determined by cytosolic Ca^2+^ measurement and cationic current recordings. The responses to mechanical forces were graded within the extracellular osmolarity range of 210–270 mOsm or pipette suction pressure range of 20–60 mmHg. The TRPC5 response to hypotonic membrane stretch is independent of phospholipase C, which is regarded as an upstream activator of TRPC channels. Gadolinium, which blocks a wide variety of cation channels including some mechanosensitive channels [20, 39], failed to block the hypotonic activation of TRPC5 but rather caused further stimulation of TRPC5-mediated [Ca^2+^]_i_ rise. We also showed that intact actin filaments are essential to both the hypotonic activation and pipette pressure-activated single-channel response of TRPC5.

Previous studies showed that hypotonic treatment could elicit a [Ca^2+^]_i_ rise in ~25% of TRPC5-transfected HEK293 cells [25, 31]. When the cells were co-transfected with type 1 histamine receptors, the hypoosmolarity-responding cells increased to ~60% [31]. However, in the present study, even without any receptor co-transfection, hypoosmotic cell swelling could evoke a [Ca^2+^]_i_ rise (F_1_/F_0_ ≥1.2) in 81 ±6% of TRPC5-transfected HEK293 cells. The reason for discrepancy is unclear. However, although Gomis *et al*. and we utilized similar clone (mouse TRPC5) and expression system (HEK293 cells), one major difference is that we stably expressed TRPC5 while Gomis *et al*. transiently expressed the channel. As will be further discussed below, mechanosensitivity of TRPC5 may involve tethering of TRPC5 to actin filament. It is possible that stable expression would allow better tethering interaction between TRPC5 and actin filaments to result in more effective mechanical response. In addition, stably transfected cells may have a higher plasma membrane localization of TRPC5 compared to transiently transfected cells.

Although we and others have shown hypoosmotic cell swelling activates TRPC5 based on [Ca^2+^]_i_ measurement and whole-cell patch clamp recording [25, 31], our current study is the first to demonstrate TRPC5 mechanosensitivity at single-channel level. Because HEK293 cells are known to express endogenous stretch-sensitive channels [50], we chose to express TRPC5 in CHO-K1 cells to study its mechanosensitivity at single-channel level. We applied negative pressure (suction) through the patch pipette, which exerts mechanical stress onto the patched membrane [44, 45], and recorded single-channel activity. The results showed that negative pipette pressure could activate a 39 pS channel in TRPC5-overexpressing CHO-K1 cells but not in control cells that were transfected with empty vector. The channel activity increased when the negative pipette pressure reached 40 mmHg or higher. When pipette pressure returned back to 0 mmHg, the channel activity diminished. The stretch activity of the channel could be inhibited by a TRPC5 blocking antibody T5E3. In addition, the stretch response persisted even when cytosolic Ca^2+^, which potentiates TRPC5 at high concentration [37], was buffered by BAPTA, indicating the stretch response is not secondary to Ca^2+^ fluctuation. These data collectively demonstrated the mechanosensitivity of TRPC5 at single-channel level.

There are several general mechanisms for mechanical activation of an ion channel. These include 1) direct channel activation by altering bilayer tension/bending/thickness, 2) indirect channel activation via mechanosensitive signaling molecules, and 3) direct channel activation by tethering to cytoskeletal elements that are exposed to mechanical forces [4, 51]. Some mechanosensitive channels, including *Drosophila* NompC and *C*. *elegans* MEC-4/MEC-10, haven been demonstrated to be gated by tethered cytoskeletons [7, 52–54]. Thus, we tested the model of tethering cytoskeletons. Our data showed that disruption of actin filaments with cytochalasin D abolished the hypoosmolarity-induced [Ca^2+^]_i_ rise in TRPC5-overexpressing HEK293 cells, supporting that mechanical force may activate TRPC5 via tethered action filaments. The detailed mechanism of interaction between TRPC5 and actin filaments interact is unclear. Previous report showed that TRPC5 may be physically coupled to the cytoskeletal-anchoring protein NHERF/EBP50 via the C-terminal PDZ-binding domain [48, 49]. However, we found that removing the C-terminal 9 amino acid residues that contain the PDZ-binding motif (VTTRL) of TRPC5 did not affect the channel response to hypoosmolarity (Fig 4G and 4H), suggesting that actin regulates TRPC5 sensitivity to mechanical stress via channel domain(s) other than the PDZ-binding motif.

Because many TRPC channels are activated by GPCR-PLC signaling pathway [11, 35], we also examined the involvement of PLC. However, inhibition of PLC with U73122 had no effect on hypoosmolarity-induced [Ca^2+^]_i_ rise in TRPC5-expressing HEK293 cells, suggesting that PLC pathway was not involved in the process. However, a recent study has shown that G_i_ proteins can directly regulate TRPC5 activity independently of PLC [55], and thus we cannot completely exclude the possibility of PLC-independent mechanism on hypoosmotic activation of TRPC5.

We did not test the model of direct channel activation by membrane tension/bending/thickness, because this would require expression of purified TRPC5 proteins in artificial lipid bilayers. In this regard, we and others have previously shown that TRPC5 can be activated by genistein, lysophosphatidylcholine and sphingosine 1-phosphate, all of which are capable of inserting into lipid bilayers, thereby altering the curvature and thickness of lipid bilayers [22–24, 56]. These previous data suggest that activity of TRPC5 may be also modulated by membrane tension/bending/thickness. Nevertheless, our present data clearly showed that cytochalasin D inhibited both the hypotonicity-induced [Ca^2+^]_i_ rise and the single-channel stretch response, suggesting that mechanical stress of the membrane is conveyed to TRPC5 via actin filaments. Further study is warranted to examine how actin filaments sense the stress and interact with TRPC5, and whether actin cytoskeletons are involved in other modes of TRPC5 activation.

TRPC5 expression has been found in many cell types with inherit mechanosensitive Ca^2+^ influx, including endothelial cells, smooth muscle cells, cardiac myocytes and arterial baroreceptor neurons [21, 57–59]. While TRPC5 knockout mice showed defective innate fear response [60], tissue-specific mechanotransduction functions in these mice remain to be uncovered. One study reported that TRPC5 knockout mice showed lower response to Von Frey fiber stimulations on paw skin mechanosensitivity [61]. The results from the current study add to the growing molecular evidence that TRPC5 may participate in the mechanotransduction of membrane stress. However, deeper and extended functional studies are necessary to ascribe a general role for TRPC5 in mechanotransduction *in vivo*.

## Supporting Information

S1 Fig Hypoosmolarity induces cell swelling and [Ca^2+^]_i_ rise in TRPC5-HEK (related to Fig 1). (A) representative image of an immunoblot comparing TRPC5 expression in HEK293 cells (left panels) and CHO-K1 cells (right panels) transfected with empty vector (*vector*) or TRPC5 (*TRPC5*). Blot with anti-β-tubulin antibody confirmed equal loading of proteins (lower fpanel). (B) bright-field time-course images showing morphological change of HEK293 cells then bath solution was exchanged from isotonic (300 mOsm) to hypotonic (210 mOsm). Scale bar represents 20 μm. The yellow-boxed area was magnified and shown on the lower panel at the respective time point. (C) morphology (upper panel) and [Ca^2+^]_i_ Fluo-3 fluorescence (lower panel) of TRPC5-HEK cells in isotonic (*300 mOsm*) and hypotonic (*210 mOsm*) bath solutions. Scale bar is 20 μm.
(TIF)

S2 Fig Hypoosmolarity increases Ca^2+^ influx and whole-cell currents through TRPC5 (related to Fig 1). (A) representative time-series traces showing [Ca^2+^]_i_ responses to hypotonicity (210 mOsm) in HEK293 cells stably transfected with vector pcDNA3. Carbachol (Cch, 100 μM) was added to show intact Ca^2+^ store mobilization. (B) representative time-series traces showing [Ca^2+^]_i_ change in TRPC5-HEK in response to hypoosmolarity when Ca^2+^ store was depleted. Thapsigargin (5 μM; *TG*) was added at the time indicated by *arrow* to deplete the ER Ca^2+^ store. (C) representative time-series traces showing [Ca^2+^]_i_ change in TRPC6-expressing HEK293 cells in response to hypoosmolarity (210 mOsm) and 1-oleoyl-acetyl-sn-glycerol (100 μm; *OAG*). OAG is a direct agonist on TRPC6. (D) representative time-series traces showing whole-cell current change of a TRPC5-HEK cell in response to hypotonicity (240 mOsm) at holding potentials of +60 mV (*open circle*) and -60 mV (*close circle*).
(TIF)

S3 Fig Gadolinium does not inhibit TRPC5 response to hypoosmolarity. (A) representative time-series trace showing [Ca^2+^]_i_ change of TRPC5-expresssing HEK293 cells in response to hypoosmolarity (210 mOsm). Traces show cells bathed in nominally Ca^2+^ free solution (0 Ca^2+^, *black trace*), treated with 4 μg/ml T5E3 (*red trace*), or with the addition of 20 μM GdCl_3_ at the time indicated by *arrow* (*blue trace*). (B) quantification of [Ca^2+^]_i_ response of TRPC5-expressing HEK293 cells at 210 mOsm, before and after addition of 20 μM GdCl_3_. Values are normalized to that before addition.
(TIF)

S4 Fig TRPC5 activity in response to pressure in excised patch recordings. A representative trace (n = 3) showing a channel preserving pressure-sensitivity in excised inside-out patch. The patch was excised from TRPC5-expressing CHO-K1 cell previously held at -60 mV at cell-attached mode with NPSS as bath solution. Arrows indicate the application and release of -40 mmHg pipette pressure.
(TIF)

## References

[1] Cantor RS. The lateral pressure profile in membranes: a physical mechanism of general anesthesia. Biochemistry. 1997;36(9):2339–44. Epub 1997/03/04. 10.1021/bi9627323 bi9627323 [pii]. . 9054538 10.1021/bi9627323

[2] Hamill OP, Martinac B. Molecular basis of mechanotransduction in living cells. Physiol Rev. 2001;81(2):685–740. Epub 2001/03/29. . 11274342 10.1152/physrev.2001.81.2.685

[3] Kung C. A possible unifying principle for mechanosensation. Nature. 2005;436(7051):647–54. Epub 2005/08/05. nature03896 [pii] 10.1038/nature03896. . 16079835 10.1038/nature03896

[4] Pedersen SF, Nilius B. Transient receptor potential channels in mechanosensing and cell volume regulation. Methods Enzymol. 2007;428:183–207. Epub 2007/09/19. S0076-6879(07)28010-3 [pii] 10.1016/S0076-6879(07)28010-3. . 17875418 10.1016/S0076-6879(07)28010-3

[5] Arnadottir J, Chalfie M. Eukaryotic mechanosensitive channels. Annu Rev Biophys. 2010;39:111–37. Epub 2010/03/03. 10.1146/annurev.biophys.37.032807.125836 . 20192782

[6] Denis V, Cyert MS. Internal Ca(2+) release in yeast is triggered by hypertonic shock and mediated by a TRP channel homologue. J Cell Biol. 2002;156(1):29–34. Epub 2002/01/10. 10.1083/jcb.200111004 jcb.200111004 [pii]. ; . 11781332 10.1083/jcb.200111004 PMC2173594

[7] O'Hagan R, Chalfie M, Goodman MB. The MEC-4 DEG/ENaC channel of Caenorhabditis elegans touch receptor neurons transduces mechanical signals. Nat Neurosci. 2005;8(1):43–50. Epub 2004/12/08. nn1362 [pii] 10.1038/nn1362. . 15580270 10.1038/nn1362

[8] Dedman A, Sharif-Naeini R, Folgering JH, Duprat F, Patel A, Honore E. The mechano-gated K(2P) channel TREK-1. Eur Biophys J. 2009;38(3):293–303. Epub 2008/03/29. 10.1007/s00249-008-0318-8 . 18369610

[9] Coste B, Xiao B, Santos JS, Syeda R, Grandl J, Spencer KS, et al Piezo proteins are pore-forming subunits of mechanically activated channels. Nature. 2012;483(7388):176–81. Epub 2012/02/22. nature10812 [pii] 10.1038/nature10812. ; 10.1038/nature10812 22343900 PMC3297710

[10] Kim SE, Coste B, Chadha A, Cook B, Patapoutian A. The role of Drosophila Piezo in mechanical nociception. Nature. 2012;483(7388):209–12. Epub 2012/02/22. nature10801 [pii] 10.1038/nature10801. ; 10.1038/nature10801 22343891 PMC3297676

[11] Venkatachalam K, Montell C. TRP channels. Annu Rev Biochem. 2007;76:387–417. Epub 2007/06/21. 10.1146/annurev.biochem.75.103004.142819 . 17579562 PMC4196875

[12] Birder LA, Nakamura Y, Kiss S, Nealen ML, Barrick S, Kanai AJ, et al Altered urinary bladder function in mice lacking the vanilloid receptor TRPV1. Nat Neurosci. 2002;5(9):856–60. Epub 2002/08/06. 10.1038/nn902nn902 [pii]. . 12161756 10.1038/nn902

[13] Earley S, Waldron BJ, Brayden JE. Critical role for transient receptor potential channel TRPM4 in myogenic constriction of cerebral arteries. Circ Res. 2004;95(9):922–9. Epub 2004/10/09. 01.RES.0000147311.54833.03 [pii] 10.1161/01.RES.0000147311.54833.03. . 15472118 10.1161/01.RES.0000147311.54833.03

[14] Mihara H, Suzuki N, Yamawaki H, Tominaga M, Sugiyama T. TRPV2 ion channels expressed in inhibitory motor neurons of gastric myenteric plexus contribute to gastric adaptive relaxation and gastric emptying in mice. Am J Physiol Gastrointest Liver Physiol. 2013;304(3):G235–40. Epub 2012/12/04. ajpgi.00256.2012 [pii] 10.1152/ajpgi.00256.2012. 10.1152/ajpgi.00256.2012 23203157

[15] Son AR, Yang YM, Hong JH, Lee SI, Shibukawa Y, Shin DM. Odontoblast TRP channels and thermo/mechanical transmission. J Dent Res. 2009;88(11):1014–9. Epub 2009/10/16. 88/11/1014 [pii] 10.1177/0022034509343413. 10.1177/0022034509343413 19828889

[16] Garrison SR, Dietrich A, Stucky CL. TRPC1 contributes to light-touch sensation and mechanical responses in low-threshold cutaneous sensory neurons. J Neurophysiol. 2012;107(3):913–22. Epub 2011/11/11. jn.00658.2011 [pii] 10.1152/jn.00658.2011. ; 10.1152/jn.00658.2011 22072513 PMC3289471

[17] Kwan KY, Allchorne AJ, Vollrath MA, Christensen AP, Zhang DS, Woolf CJ, et al TRPA1 contributes to cold, mechanical, and chemical nociception but is not essential for hair-cell transduction. Neuron. 2006;50(2):277–89. Epub 2006/04/25. S0896-6273(06)00269-8 [pii] 10.1016/j.neuron.2006.03.042. . 16630838 10.1016/j.neuron.2006.03.042

[18] Liedtke W, Friedman JM. Abnormal osmotic regulation in trpv4-/- mice. Proc Natl Acad Sci U S A. 2003;100(23):13698–703. Epub 2003/10/29. 10.1073/pnas.17354161001735416100 [pii]. ; . 14581612 10.1073/pnas.1735416100 PMC263876

[19] Welsh DG, Morielli AD, Nelson MT, Brayden JE. Transient receptor potential channels regulate myogenic tone of resistance arteries. Circ Res. 2002;90(3):248–50. Epub 2002/02/28. . 11861411 10.1161/hh0302.105662

[20] Sukharev S, Sachs F. Molecular force transduction by ion channels: diversity and unifying principles. J Cell Sci. 2012;125(Pt 13):3075–83. Epub 2012/07/17. jcs.092353 [pii] 10.1242/jcs.092353. ; 10.1242/jcs.092353 22797911 PMC3434843

[21] Glazebrook PA, Schilling WP, Kunze DL. TRPC channels as signal transducers. Pflugers Arch. 2005;451(1):125–30. Epub 2005/06/23. 10.1007/s00424-005-1468-5 . 15971079

[22] Beech DJ. Bipolar phospholipid sensing by TRPC5 calcium channel. Biochem Soc Trans. 2007;35(Pt 1):101–4. Epub 2007/01/20. BST0350101 [pii] 10.1042/BST0350101. . 17233612 10.1042/BST0350101

[23] Wong CO, Huang Y, Yao X. Genistein potentiates activity of the cation channel TRPC5 independently of tyrosine kinases. Br J Pharmacol. 2010;159(7):1486–96. Epub 2010/03/18. BPH636 [pii] 10.1111/j.1476-5381.2010.00636.x. ; 10.1111/j.1476-5381.2010.00636.x 20233211 PMC2850405

[24] Raghunathan M, Zubovski Y, Venable RM, Pastor RW, Nagle JF, Tristram-Nagle S. Structure and elasticity of lipid membranes with genistein and daidzein bioflavinoids using X-ray scattering and MD simulations. J Phys Chem B. 2012;116(13):3918–27. Epub 2012/02/14. 10.1021/jp211904j ; . 22324769 PMC3320743

[25] Gomis A, Soriano S, Belmonte C, Viana F. Hypoosmotic- and pressure-induced membrane stretch activate TRPC5 channels. J Physiol. 2008;586(Pt 23):5633–49. Epub 2008/10/04. jphysiol.2008.161257 [pii] 10.1113/jphysiol.2008.161257. 10.1113/jphysiol.2008.161257 18832422 PMC2655392

[26] Xu SZ, Zeng F, Lei M, Li J, Gao B, Xiong C, et al Generation of functional ion-channel tools by E3 targeting. Nat Biotechnol. 2005;23(10):1289–93. Epub 2005/09/20. nbt1148 [pii] 10.1038/nbt1148. . 16170312 10.1038/nbt1148

[27] Auerbach A. Single-channel dose-response studies in single, cell-attached patches. Biophys J. 1991;60(3):660–70. Epub 1991/09/01. S0006-3495(91)82095-1 [pii] 10.1016/S0006-3495(91)82095-1. ; . 1718468 10.1016/S0006-3495(91)82095-1 PMC1260109

[28] Ohya Y, Adachi N, Nakamura Y, Setoguchi M, Abe I, Fujishima M. Stretch-activated channels in arterial smooth muscle of genetic hypertensive rats. Hypertension. 1998;31(1 Pt 2):254–8. Epub 1998/02/07. . 9453312 10.1161/01.hyp.31.1.254

[29] Dai J, Sheetz MP, Wan X, Morris CE. Membrane tension in swelling and shrinking molluscan neurons. J Neurosci. 1998;18(17):6681–92. Epub 1998/08/26. . 9712640 10.1523/JNEUROSCI.18-17-06681.1998 PMC6792972

[30] Diz-Munoz A, Fletcher DA, Weiner OD. Use the force: membrane tension as an organizer of cell shape and motility. Trends Cell Biol. 2013;23(2):47–53. Epub 2012/11/06. S0962-8924(12)00177-8 [pii] 10.1016/j.tcb.2012.09.006. ; 10.1016/j.tcb.2012.09.006 23122885 PMC3558607

[31] Jemal I, Soriano S, Conte AL, Morenilla C, Gomis A. G protein-coupled receptor signalling potentiates the osmo-mechanical activation of TRPC5 channels. Pflugers Arch. 2014;466(8):1635–46. Epub 2013/11/02. 10.1007/s00424-013-1392-z . 24177920

[32] Jung S, Muhle A, Schaefer M, Strotmann R, Schultz G, Plant TD. Lanthanides potentiate TRPC5 currents by an action at extracellular sites close to the pore mouth. J Biol Chem. 2003;278(6):3562–71. Epub 2002/11/29. 10.1074/jbc.M211484200M211484200 [pii]. . 12456670 10.1074/jbc.M211484200

[33] Alexander SP, Mathie A, Peters JA. Guide to Receptors and Channels (GRAC), 5th edition. Br J Pharmacol. 2011;164 Suppl 1:S1–324. Epub 2011/11/09. 10.1111/j.1476-5381.2011.01649_1.x ; . 22040146 PMC3315626

[34] Xu SZ, Zeng F, Boulay G, Grimm C, Harteneck C, Beech DJ. Block of TRPC5 channels by 2-aminoethoxydiphenyl borate: a differential, extracellular and voltage-dependent effect. Br J Pharmacol. 2005;145(4):405–14. Epub 2005/04/05. 0706197 [pii] 10.1038/sj.bjp.0706197. ; . 15806115 10.1038/sj.bjp.0706197 PMC1576154

[35] Trebak M, Lemonnier L, Smyth JT, Vazquez G, Putney JW Jr. Phospholipase C-coupled receptors and activation of TRPC channels. Handb Exp Pharmacol. 2007;(179):593–614. Epub 2007/01/16. 10.1007/978-3-540-34891-7_35 . 17217081

[36] Storch U, Mederos y Schnitzler M, Gudermann T. G protein-mediated stretch reception. Am J Physiol Heart Circ Physiol. 2012;302(6):H1241–9. Epub 2012/01/10. ajpheart.00818.2011 [pii] 10.1152/ajpheart.00818.2011. 10.1152/ajpheart.00818.2011 22227128

[37] Blair NT, Kaczmarek JS, Clapham DE. Intracellular calcium strongly potentiates agonist-activated TRPC5 channels. J Gen Physiol. 2009;133(5):525–46. Epub 2009/04/29. jgp.200810153 [pii] 10.1085/jgp.200810153. ; 10.1085/jgp.200810153 19398778 PMC2712973

[38] Schaefer M, Plant TD, Obukhov AG, Hofmann T, Gudermann T, Schultz G. Receptor-mediated regulation of the nonselective cation channels TRPC4 and TRPC5. J Biol Chem. 2000;275(23):17517–26. Epub 2000/06/06. 275/23/17517 [pii]. . 10837492 10.1074/jbc.275.23.17517

[39] Ermakov YA, Kamaraju K, Sengupta K, Sukharev S. Gadolinium ions block mechanosensitive channels by altering the packing and lateral pressure of anionic lipids. Biophys J. 2010;98(6):1018–27. Epub 2010/03/23. S0006-3495(09)06004-4 [pii] 10.1016/j.bpj.2009.11.044. ; 10.1016/j.bpj.2009.11.044 20303859 PMC2849073

[40] Carr MJ, Gover TD, Weinreich D, Undem BJ. Inhibition of mechanical activation of guinea-pig airway afferent neurons by amiloride analogues. Br J Pharmacol. 2001;133(8):1255–62. Epub 2001/08/11. 10.1038/sj.bjp.0704197 ; . 11498511 PMC1621149

[41] Andresen MC, Yang M. Gadolinium and mechanotransduction of rat aortic baroreceptors. Am J Physiol. 1992;262(5 Pt 2):H1415–21. Epub 1992/05/01. . 1590446 10.1152/ajpheart.1992.262.5.H1415

[42] Lin YW, Cheng CM, Leduc PR, Chen CC. Understanding sensory nerve mechanotransduction through localized elastomeric matrix control. PLoS One. 2009;4(1):e4293 Epub 2009/01/29. 10.1371/journal.pone.0004293 ; . 19173000 PMC2627935

[43] Yoshimura K, Sokabe M. Mechanosensitivity of ion channels based on protein-lipid interactions. J R Soc Interface. 2010;7 Suppl 3:S307–20. Epub 2010/04/02. rsif.2010.0095.focus [pii] 10.1098/rsif.2010.0095.focus. ; 10.1098/rsif.2010.0095.focus 20356872 PMC2943882

[44] Opsahl LR, Webb WW. Lipid-glass adhesion in giga-sealed patch-clamped membranes. Biophys J. 1994;66(1):75–9. Epub 1994/01/01. S0006-3495(94)80752-0 [pii] 10.1016/S0006-3495(94)80752-0. ; . 8130347 10.1016/S0006-3495(94)80752-0 PMC1275665

[45] Ursell T, Agrawal A, Phillips R. Lipid bilayer mechanics in a pipette with glass-bilayer adhesion. Biophys J. 2011;101(8):1913–20. Epub 2011/10/19. S0006-3495(11)01079-4 [pii] 10.1016/j.bpj.2011.08.057. ; 10.1016/j.bpj.2011.08.057 22004745 PMC3192956

[46] Strubing C, Krapivinsky G, Krapivinsky L, Clapham DE. TRPC1 and TRPC5 form a novel cation channel in mammalian brain. Neuron. 2001;29(3):645–55. Epub 2001/04/13. S0896-6273(01)00240-9 [pii]. . 11301024 10.1016/s0896-6273(01)00240-9

[47] Berrier C, Besnard M, Ajouz B, Coulombe A, Ghazi A. Multiple mechanosensitive ion channels from Escherichia coli, activated at different thresholds of applied pressure. J Membr Biol. 1996;151(2):175–87. Epub 1996/05/01. . 8661505 10.1007/s002329900068

[48] Tang Y, Tang J, Chen Z, Trost C, Flockerzi V, Li M, et al Association of mammalian trp4 and phospholipase C isozymes with a PDZ domain-containing protein, NHERF. J Biol Chem. 2000;275(48):37559–64. Epub 2000/09/12. 10.1074/jbc.M006635200M006635200 [pii]. . 10980202 10.1074/jbc.M006635200

[49] Obukhov AG, Nowycky MC. TRPC5 activation kinetics are modulated by the scaffolding protein ezrin/radixin/moesin-binding phosphoprotein-50 (EBP50). J Cell Physiol. 2004;201(2):227–35. Epub 2004/08/31. 10.1002/jcp.20057 . 15334657

[50] Maroto R, Raso A, Wood TG, Kurosky A, Martinac B, Hamill OP. TRPC1 forms the stretch-activated cation channel in vertebrate cells. Nat Cell Biol. 2005;7(2):179–85. Epub 2005/01/25. ncb1218 [pii] 10.1038/ncb1218. . 15665854 10.1038/ncb1218

[51] Christensen AP, Corey DP. TRP channels in mechanosensation: direct or indirect activation? Nat Rev Neurosci. 2007;8(7):510–21. Epub 2007/06/23. nrn2149 [pii] 10.1038/nrn2149. . 17585304 10.1038/nrn2149

[52] Arnadottir J, O'Hagan R, Chen Y, Goodman MB, Chalfie M. The DEG/ENaC protein MEC-10 regulates the transduction channel complex in Caenorhabditis elegans touch receptor neurons. J Neurosci. 2011;31(35):12695–704. Epub 2011/09/02. 31/35/12695 [pii] 10.1523/JNEUROSCI.4580-10.2011. ; 10.1523/JNEUROSCI.4580-10.2011 21880930 PMC3172708

[53] Cheng LE, Song W, Looger LL, Jan LY, Jan YN. The role of the TRP channel NompC in Drosophila larval and adult locomotion. Neuron. 2010;67(3):373–80. Epub 2010/08/11. S0896-6273(10)00542-8 [pii] 10.1016/j.neuron.2010.07.004. ; 10.1016/j.neuron.2010.07.004 20696376 PMC2933178

[54] Yan Z, Zhang W, He Y, Gorczyca D, Xiang Y, Cheng LE, et al Drosophila NOMPC is a mechanotransduction channel subunit for gentle-touch sensation. Nature. 2013;493(7431):221–5. Epub 2012/12/12. nature11685 [pii] 10.1038/nature11685. 10.1038/nature11685 23222543 PMC3917554

[55] Jeon JP, Hong C, Park EJ, Jeon JH, Cho NH, Kim IG, et al Selective Galphai subunits as novel direct activators of transient receptor potential canonical (TRPC)4 and TRPC5 channels. J Biol Chem. 2012;287(21):17029–39. Epub 2012/03/30. M111.326553 [pii] 10.1074/jbc.M111.326553. ; 10.1074/jbc.M111.326553 22457348 PMC3366817

[56] McMahon HT, Gallop JL. Membrane curvature and mechanisms of dynamic cell membrane remodelling. Nature. 2005;438(7068):590–6. Epub 2005/12/02. nature04396 [pii] 10.1038/nature04396. . 16319878 10.1038/nature04396

[57] Bush EW, Hood DB, Papst PJ, Chapo JA, Minobe W, Bristow MR, et al Canonical transient receptor potential channels promote cardiomyocyte hypertrophy through activation of calcineurin signaling. J Biol Chem. 2006;281(44):33487–96. Epub 2006/09/05. M605536200 [pii] 10.1074/jbc.M605536200. . 16950785 10.1074/jbc.M605536200

[58] Flemming PK, Dedman AM, Xu SZ, Li J, Zeng F, Naylor J, et al Sensing of lysophospholipids by TRPC5 calcium channel. J Biol Chem. 2006;281(8):4977–82. Epub 2005/12/22. M510301200 [pii] 10.1074/jbc.M510301200. . 16368680 10.1074/jbc.M510301200

[59] Yip H, Chan WY, Leung PC, Kwan HY, Liu C, Huang Y, et al Expression of TRPC homologs in endothelial cells and smooth muscle layers of human arteries. Histochem Cell Biol. 2004;122(6):553–61. Epub 2004/11/13. 10.1007/s00418-004-0720-y. . 15538613 10.1007/s00418-004-0720-y

[60] Riccio A, Li Y, Moon J, Kim KS, Smith KS, Rudolph U, et al Essential role for TRPC5 in amygdala function and fear-related behavior. Cell. 2009;137(4):761–72. Epub 2009/05/20. S0092-8674(09)00376-6 [pii] 10.1016/j.cell.2009.03.039. ; 10.1016/j.cell.2009.03.039 19450521 PMC2719954

[61] Zimmermann K, Lennerz JK, Hein A, Link AS, Kaczmarek JS, Delling M, et al Transient receptor potential cation channel, subfamily C, member 5 (TRPC5) is a cold-transducer in the peripheral nervous system. Proc Natl Acad Sci U S A. 2011;108(44):18114–9. Epub 2011/10/26. 1115387108 [pii] 10.1073/pnas.1115387108. ; 10.1073/pnas.1115387108 22025699 PMC3207667

