# Supplementary figures and images for: Plasma Membrane Mechanical Stress Activates TRPC5 Channels
Source: PLoS One. 2015 Apr 7;10(4):e0122227. doi: 10.1371/journal.pone.0122227 (PMC4388645; doi:10.1371/journal.pone.0122227)

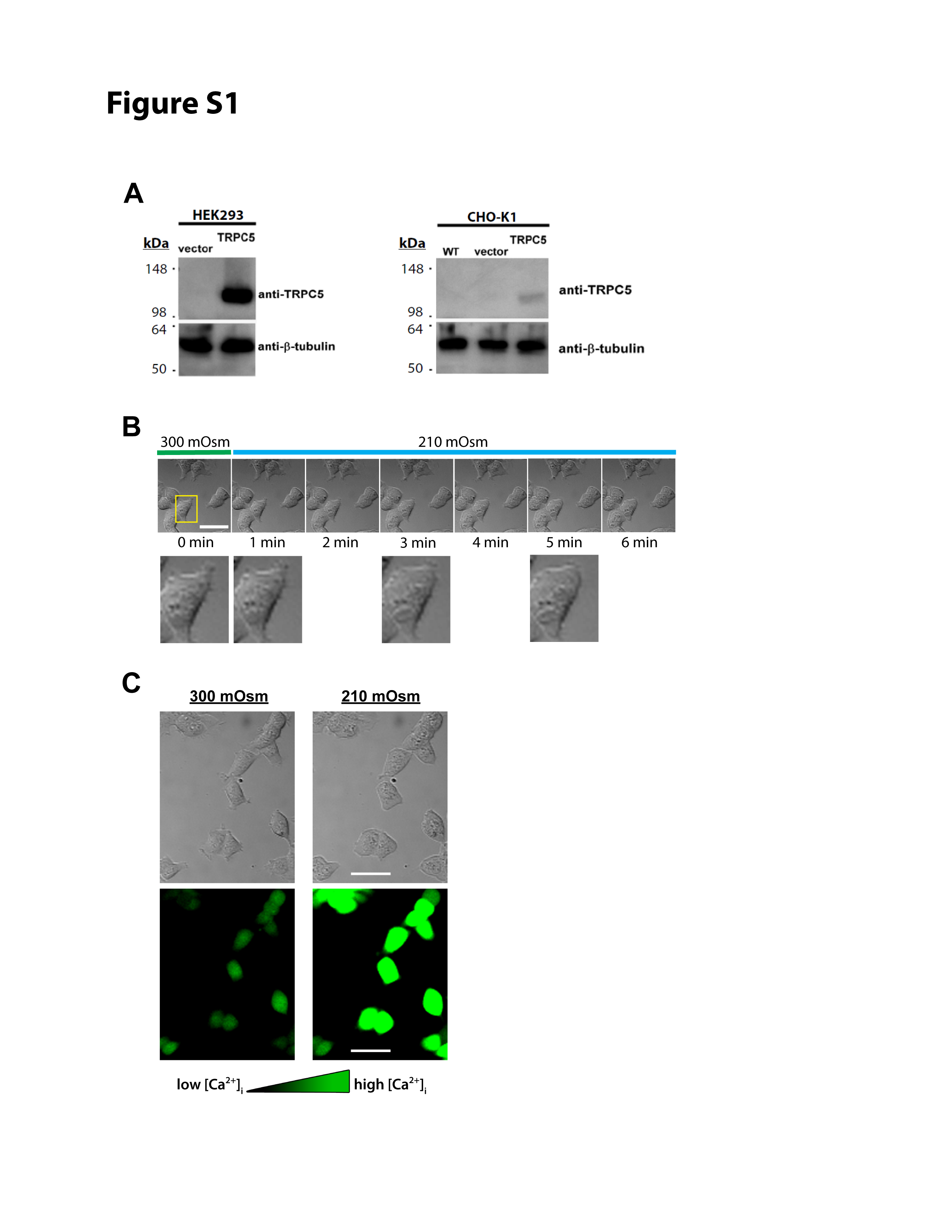

Supplement: S1 Fig — (A) representative image of an immunoblot comparing TRPC5 expression in HEK293 cells (left panels) and CHO-K1 cells (right panels) transfected with empty vector (vector) or TRPC5 (TRPC5). Blot with anti-β-tubulin antibody confirmed equal loading of proteins (lower fpanel). (B) bright-field time-course images showing morphological change of HEK293 cells then bath solution was exchanged from isotonic (300 mOsm) to hypotonic (210 mOsm). Scale bar represents 20 μm. The yellow-boxed area was magnified and shown on the lower panel at the respective time point. (C) morphology (upper panel) and [Ca2+]i Fluo-3 fluorescence (lower panel) of TRPC5-HEK cells in isotonic (300 mOsm) and hypotonic (210 mOsm) bath solutions. Scale bar is 20 μm. (TIF) [file pone.0122227.s001.tif]

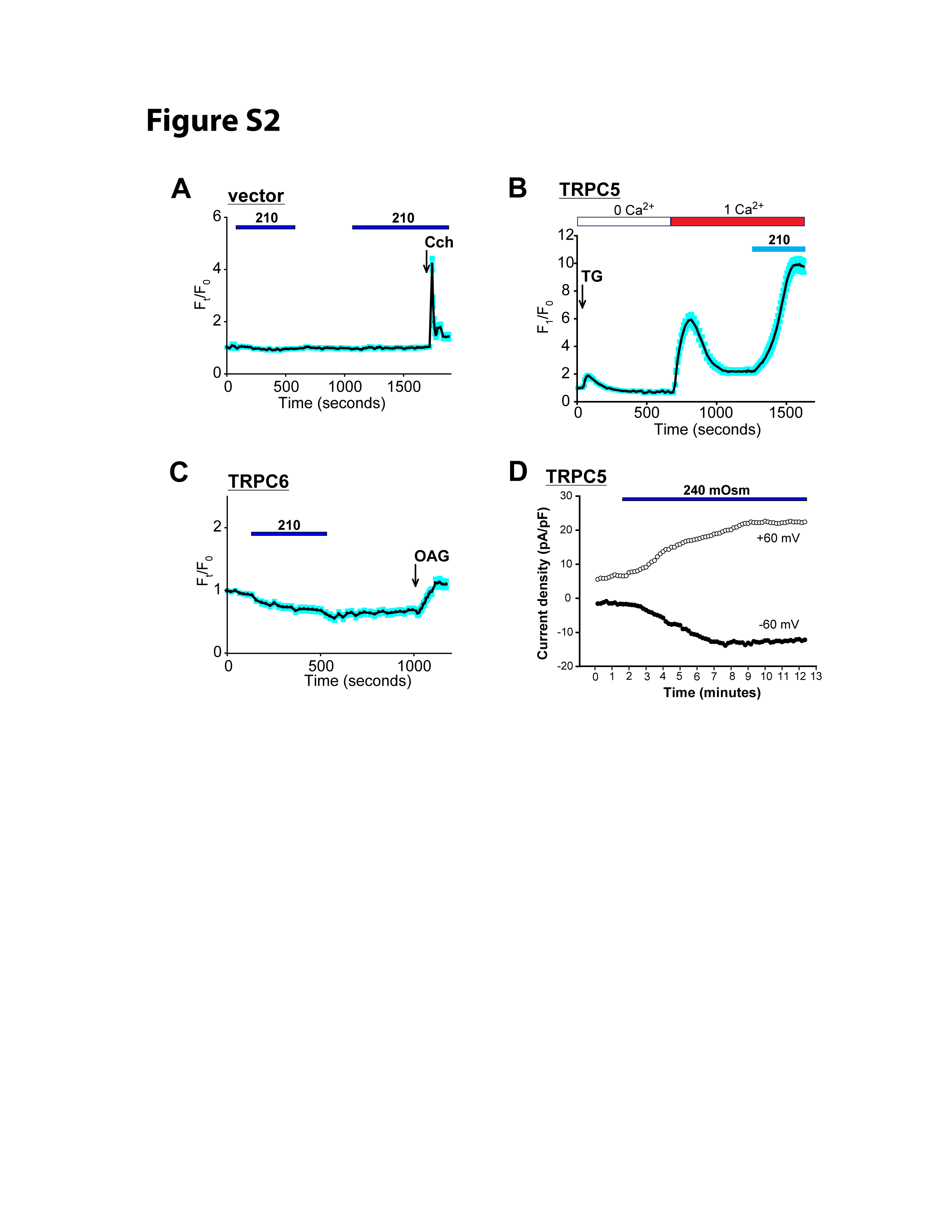

Supplement: S2 Fig — (A) representative time-series traces showing [Ca2+]i responses to hypotonicity (210 mOsm) in HEK293 cells stably transfected with vector pcDNA3. Carbachol (Cch, 100 μM) was added to show intact Ca2+ store mobilization. (B) representative time-series traces showing [Ca2+]i change in TRPC5-HEK in response to hypoosmolarity when Ca2+ store was depleted. Thapsigargin (5 μM; TG) was added at the time indicated by arrow to deplete the ER Ca2+ store. (C) representative time-series traces showing [Ca2+]i change in TRPC6-expressing HEK293 cells in response to hypoosmolarity (210 mOsm) and 1-oleoyl-acetyl-sn-glycerol (100 μm; OAG). OAG is a direct agonist on TRPC6. (D) representative time-series traces showing whole-cell current change of a TRPC5-HEK cell in response to hypotonicity (240 mOsm) at holding potentials of +60 mV (open circle) and -60 mV (close circle). (TIF) [file pone.0122227.s002.tif]

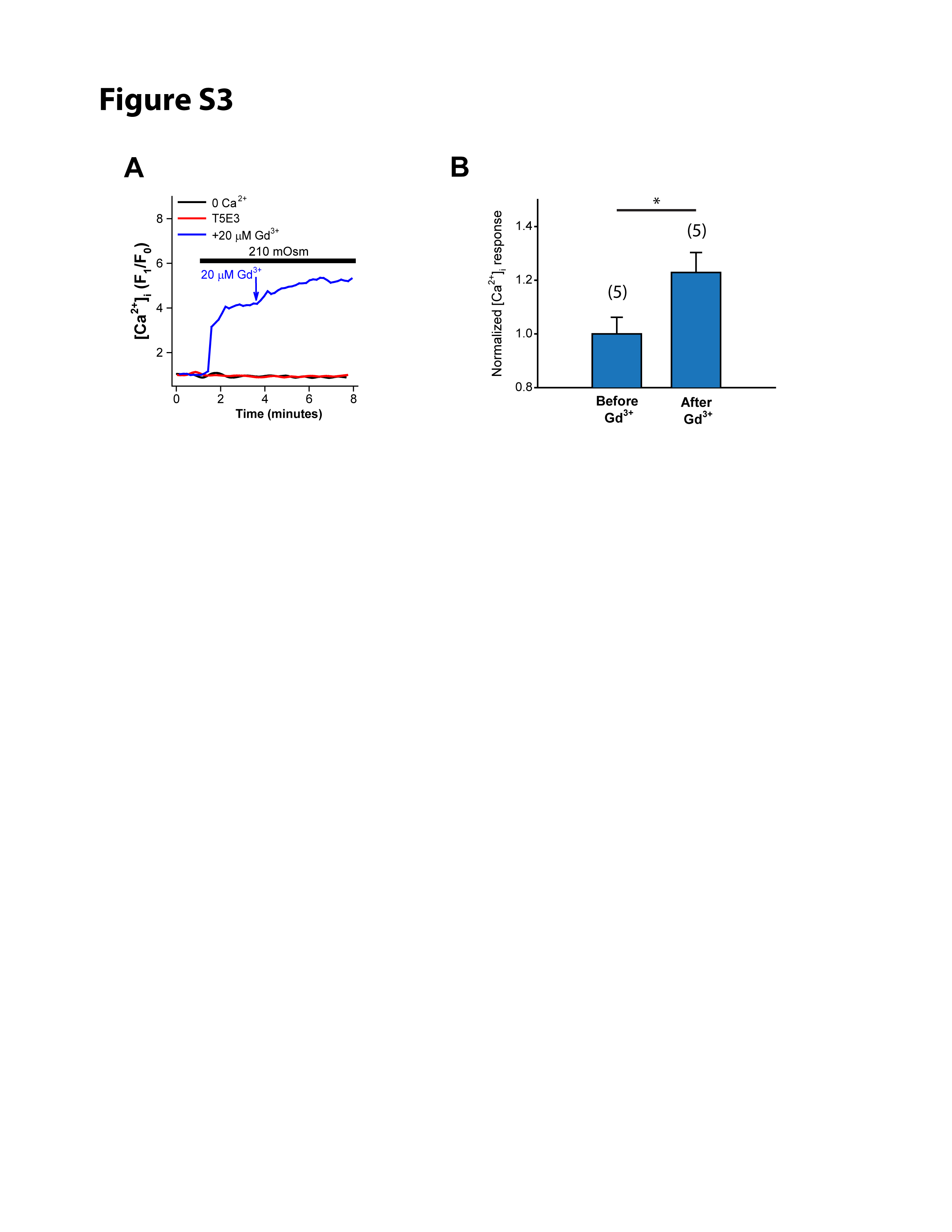

Supplement: S3 Fig — (A) representative time-series trace showing [Ca2+]i change of TRPC5-expresssing HEK293 cells in response to hypoosmolarity (210 mOsm). Traces show cells bathed in nominally Ca2+ free solution (0 Ca2+, black trace), treated with 4 μg/ml T5E3 (red trace), or with the addition of 20 μM GdCl3 at the time indicated by arrow (blue trace). (B) quantification of [Ca2+]i response of TRPC5-expressing HEK293 cells at 210 mOsm, before and after addition of 20 μM GdCl3. Values are normalized to that before addition. (TIF) [file pone.0122227.s003.tif]

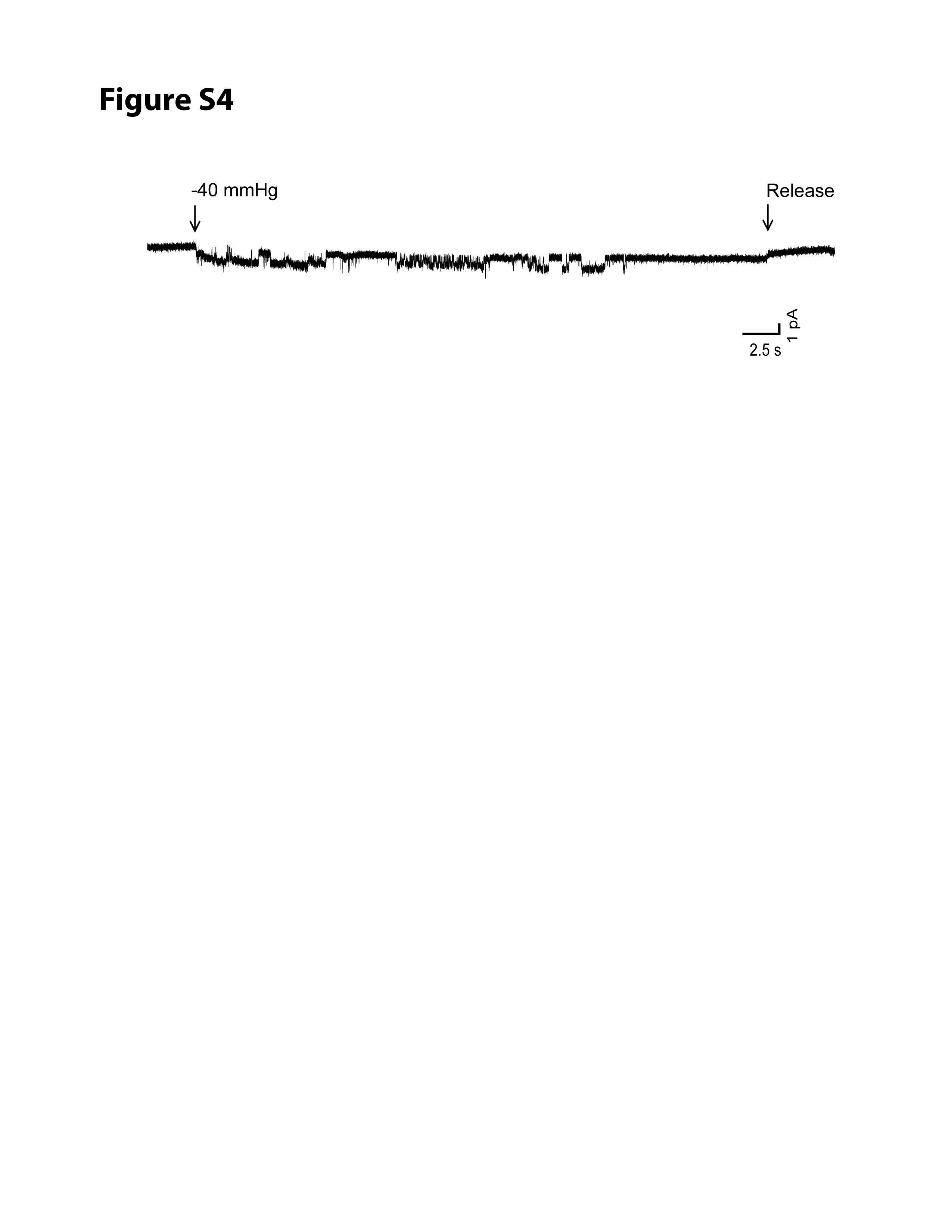

Supplement: S4 Fig — A representative trace (n = 3) showing a channel preserving pressure-sensitivity in excised inside-out patch. The patch was excised from TRPC5-expressing CHO-K1 cell previously held at -60 mV at cell-attached mode with NPSS as bath solution. Arrows indicate the application and release of -40 mmHg pipette pressure. (TIF) [file pone.0122227.s004.tif]
